# Thermal transport characterization of carbon and silicon doped stanene nanoribbon: an equilibrium molecular dynamics study

**DOI:** 10.1039/c8ra06156d

**Published:** 2018-09-12

**Authors:** Ishtiaque Ahmed Navid, Samia Subrina

**Affiliations:** Department of Electrical and Electronic Engineering, Bangladesh University of Engineering and Technology Dhaka 1205 Bangladesh samiasubrina@eee.buet.ac.bd ssubr002@ucr.edu +88-02-9668054 +880-19-3795-9083 +88-02-9668054

## Abstract

Equilibrium molecular dynamics simulation has been carried out for the thermal transport characterization of nanometer sized carbon and silicon doped stanene nanoribbon (STNR). The thermal conduction properties of doped stanene nanostructures are yet to be explored and hence in this study, we have investigated the impact of carbon and silicon doping concentrations as well as doping patterns namely single doping, double doping and edge doping on the thermal conductivity of nanometer sized zigzag STNR. The room temperature thermal conductivities of 15 nm × 4 nm doped zigzag STNR at 2% carbon and silicon doping concentration are computed to be 9.31 ± 0.33 W m^−1^ K^−1^ and 7.57 ± 0.48 W m^−1^ K^−1^, respectively whereas the thermal conductivity for the pristine STNR of the same dimension is calculated as 1.204 ± 0.21 W m^−1^ K^−1^. We find that the thermal conductivity of both carbon and silicon doped STNR increases with the increasing doping concentration for both carbon and silicon doping. The magnitude of increase in STNR thermal conductivity due to carbon doping has been found to be greater than that of silicon doping. Different doping patterns manifest different degrees of change in doped STNR thermal conductivity. Double doping pattern for both carbon and silicon doping induces the largest extent of enhancement in doped STNR thermal conductivity followed by single doping pattern and edge doping pattern respectively. The temperature and width dependence of doped STNR thermal conductivity has also been studied. For a particular doping concentration, the thermal conductivity of both carbon and silicon doped STNR shows a monotonic decaying trend at elevated temperatures while an opposite pattern is observed for width variation *i.e.* thermal conductivity increases with the increase in ribbon width. Such comprehensive study on doped stanene would encourage further investigation on the proper optimization of thermal transport characteristics of stanene nanostructures and provide deep insight in realizing the potential application of doped STNR in thermoelectric as well as thermal management of stanene based nanoelectronic devices.

## Abstract 2

Tunable thermal transport of doped stanene nanoribbon considering the impact of doping concentration, doping pattern, temperature and nanoribbon width.

## Graphical abstract

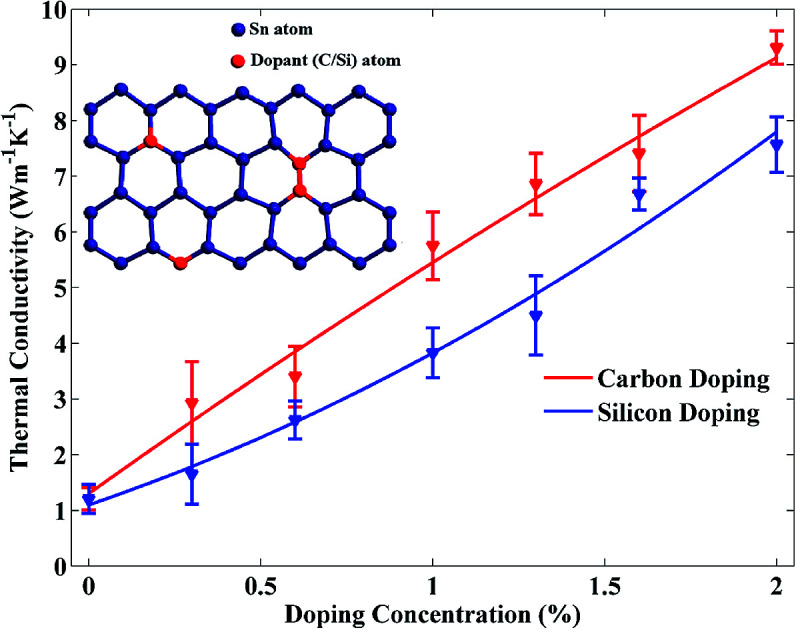

## 1. Introduction

The synthesis as well as characterization of graphene, due to its intriguing electronic,^1^ thermal^2^ and mechanical^3^ properties, has instigated enormous research interest into two dimensional (2D) nanomaterials.^4–8^ Recently, the synthesis of the 2D structures of heavier group-IV elements namely silicene, germanene and stanene^9,10^ have incited attention due to their graphene like honeycomb structure. Stanene is a 2D buckled hexagonal allotrope of tin (Sn)^11^ with enhanced thermoelectricity^12^ and quantum anomalous Hall effect.^13^ It has promising potential as a topological insulator^14^ and a topological superconductor^15^ as well as a quantum Hall insulator.^16,17^ Furthermore, spin orbiting coupling (SOC) induces a bulk bandgap of ∼0.1 eV for free-standing stanene while it shows zero bandgap without spin orbiting coupling.^11^ It has gapless edge states with band dispersion in the bulk gap as well as helical edge states with the spin-momentum locked which can be used for dissipationless conduction.^11,18^ Moreover, there are reports of very low thermal conductivity^17,19^ and high carrier mobility^20^ of pristine stanene which make stanene a promising candidate for next generation thermoelectric applications. Significant improvement in the thermoelectric figure of merit (*zT*) can be achieved in a system with simultaneously good electrical and low phonon transport. This fact urges the investigation of electrical as well as thermal transport characteristics of stanene nanostructures and explores the prospect of stanene in thermoelectric applications.

Chemical doping of materials with foreign atoms is an effective way to alter material properties. Wei *et al.* synthesized nitrogen doped graphene using chemical vapor deposition and observed that it shows n-type behavior with decreased mobility hence decreased conductivity but enhanced on/off ratio.^21^ Panchakarla *et al.* also synthesized bilayer structures of boron and nitrogen doped graphene which resulted in p-type and n-type doping respectively and reported that both types of doping caused increase in electrical conductivity in the bilayer structure.^22^ On the other hand, quantum anomalous hall effect and tunable topological states have been reported by Zhang *et al.* in 3d transitional metals doped silicene.^23^ Garg *et al.* performed density functional theory calculations and reported band gap opening in stanene with doped boron-nitride^24^ whereas Shaidu *et al.* observed superconductivity in lithium and calcium doped stanene.^25^ The doping characteristics of 31 different adatoms on monolayer stanene have also been investigated by Naqvi *et al.*^26^ On the other hand, thermal transport characterization of doped stanene is yet to be explored. However, the thermal transport in doped graphene with nitrogen^27^ and hydrogen^28^ has been studied. The tunable thermal conductivity of silicene by isotopic doping^29^ and germanium doping^30^ has also been reported. The stanene analogues of 2D hexagonal group-IV elements carbon and silicon *i.e.* graphene and silicene of nanometer size are reported to have much higher thermal conductivities^31,32^ compared to that of stanene nanostructure.^33^ The calculated thermal conductivity of 10 nm × 3 nm sized pristine stanene nanoribbon (STNR) is 0.95 W m^−1^ K^−1^.^33^ These suggest that a detail investigation on the thermal transport characteristics of doped stanene nanostructures is significant for the proper understanding of possible industrial applications of stanene nanostructures.

In this study, we perform equilibrium molecular dynamics (EMD) simulation for the calculation of the thermal conductivity of carbon and silicon doped zigzag STNR. We investigate how the carbon and silicon doping concentrations influence the STNR thermal conductivity along with the calculation of heat current autocorrelation function (HCACF) and phonon density of states (PDOS). We also carry out a comparative study on thermal transport variation in STNR due to carbon and silicon doping. Subsequently, the effect of various types of doping patterns namely single doping, double doping, and edge doping on the thermal transport of STNR has been evaluated. Finally, the impact of varying temperature as well as nanoribbon width on the thermal conductivity of doped STNR has been examined at different carbon and silicon doping concentrations.

## 2. Simulation details

**Fig. 1:**
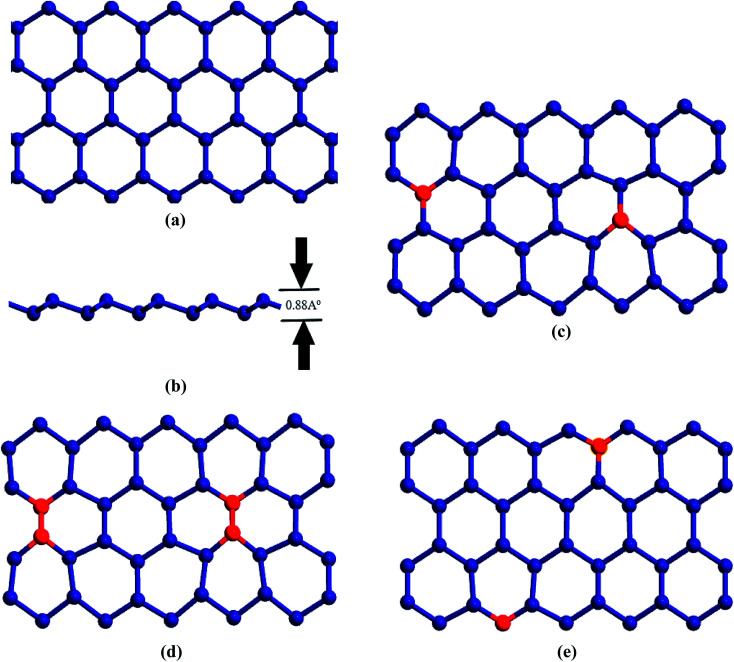
(a) Top view and (b) front view for the atomistic representation of a pristine zigzag STNR. The buckling height is shown in (b). Schematic representation of doped STNR with (c) single doping (d) double doping and (e) edge doping patterns. The tin and doped atoms are depicted by dark blue and red colored balls, respectively. Modeled structure is doped with either carbon or silicon atoms.

Fig. 1(a) and (b) are the schematic representations for the atomic structure of 15 nm × 4 nm sized pristine zigzag STNR considered in this study. Geometric optimization process was carried out involving energy minimization with steepest decent algorithm accompanied by equilibration and thermalization. The Sn–Sn bond length in the equilibrated STNR is 2.83 Å with the geometry optimized buckling height of 0.88 Å and lattice constant of 4.68 Å. These values are consistent with the reported literature values.^24,26,34–36^

We have modeled three types of doping patterns in STNR with carbon and silicon atoms of different concentration as shown in Fig. 1(c)–(e) and investigated the impact of doping in thermal transport of STNR. The single doped structures result from the random substitution of a tin atom by the dopant atom, either carbon or silicon, as represented in Fig. 1(c). Fig. 1(d) depicts the double doped structure which is generated by replacing a pair of bonding tin atoms by a pair of bonding dopant atoms. Edge doping is considered as a particular form of single doping which involves the substitution of a tin atom by the dopant atom only on the edge of the nanoribbon structure as shown in Fig. 1(e).

EMD simulations using LAMMPS (Large-scale Atomic/Molecular Massively Parallel Simulator)^37^ has been carried out in order to compute the thermal conductivity of carbon and silicon doped STNR. The Sn–Sn bond interaction in stanene has been modeled using the optimized Tersoff type bond order potential parameters proposed by Cherukara *et al.*^19^ On the other hand, for describing the C–C and Si–Si atomic interaction, optimized Tersoff and Brenner empirical potential^38^ and Stillinger–Weber (SW) potential^39^ parameters have been used, respectively. Furthermore, the Sn–C and Sn–Si bonding interactions are described by employing standard 12-6 Lennard-Jones (LJ) potential *V*(*r*) as following:1
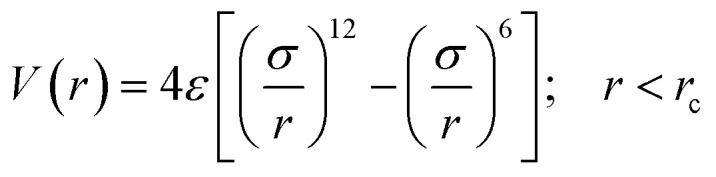
where *ε* and *σ* are energy parameter and distance parameter respectively as well as *r* and *r*_c_ are interatomic distance and cutoff distance respectively. In this work, Universal Force Field^40^ has been used for computing the LJ potential parameters. The calculated values of these parameters for tin–carbon interaction of carbon doped stanene sample are *ε*_Sn–C_ = 10.58 meV, *σ*_Sn–C_ = 3.664 Å and *r*_c,Sn–C_ = 3.5 *σ*_Sn–C_ = 12.824 Å. The same set of parameters for silicon doping have been calculated to be *ε*_Sn–Si_ = 20.69 meV, *σ*_Sn–Si_ = 3.8615 Å and *r*_c,Sn–Si_ = 3.5 *σ*_Sn–Si_ = 13.515 Å.

We applied periodic boundary condition along zigzag direction in our EMD simulation. The system energy was minimized using steepest descent algorithm and velocity-Verlet integrator was employed for the numerical integration of the equations of atomic motions with a time step of 0.5 fs. The system equilibration as well as thermalization was performed applying Nose–Hoover thermostat for 1.6 × 10^5^ time steps followed by NVE ensemble for 2 × 10^5^ time steps. Linear response theorem^41^ is applied for calculating thermal conductivity in EMD. In this case, the heat current vectors along with their correlations are computed throughout the simulation. Thermal conductivity is related to the ensemble average of HCACF by the well-known Green–Kubo formulation:2
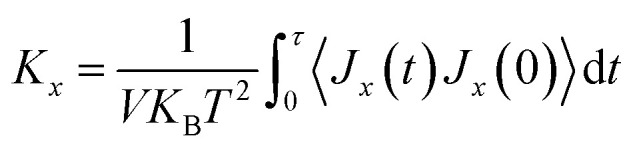
Here, *K*_*x*_ is the thermal conductivity in *x* direction, *V* is the system volume, *K*_B_ is the Boltzmann constant, *J*_*x*_(*t*) is the heat current in *x* direction and *T* is the system temperature. The STNR surface area and van der Waals thickness *i.e.* stanene interplanar separation (3.3 Å)^36,42^ are multiplied in order to compute the system volume. *τ* represents the time required for the reasonable HCACF decay termed as correlation time. The term with angular bracket of eqn (2) represents the ensemble average of the HCACF. For the implementation of eqn (2) in EMD computation, the integral term is employed as the summation of discrete terms^43,44^ shown in the following equation:3
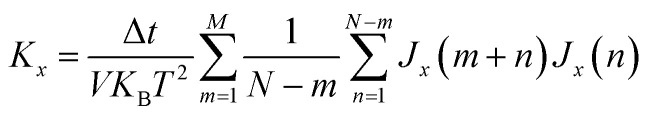
where molecular dynamics (MD) simulation time step is denoted by Δ*t*, *N* is the total number of simulation steps and *M* represents the number of time steps required for HCACF such that *M*Δ*t* corresponds to the correlation time *τ*. *J*_*x*_(*m* + *n*) and *J*_*x*_(*n*) denote the heat current in *x* direction at MD time-steps (*m* + *n*) and *n*, respectively.

We recorded the heat current data in every 5 steps in order to obtain the HCACFs. Subsequently, 10 of the obtained HCACFs were averaged for computing the heat current autocorrelation values. The thermal conductivity values were calculated applying eqn (2). Finally, the converged value of average thermal conductivity is taken as the average of 5 independent microcanonical ensembles each with a different initial velocity.

Fix Phonon command^45^ of LAMMPS has been employed for evaluating the phonon density of states (PDOS). It involves the direct calculation of the dynamical matrices from MD simulation based on fluctuation dissipation theory. Once the dynamical matrices were obtained, PDOS was calculated using an auxiliary post-processing code called ‘phana’. In this study, a tricubic^46^ interpolation method with uniform *q* (wave vector) points was taken under consideration for the calculation of PDOS.

## 3. Results and discussion

**Fig. 2:**
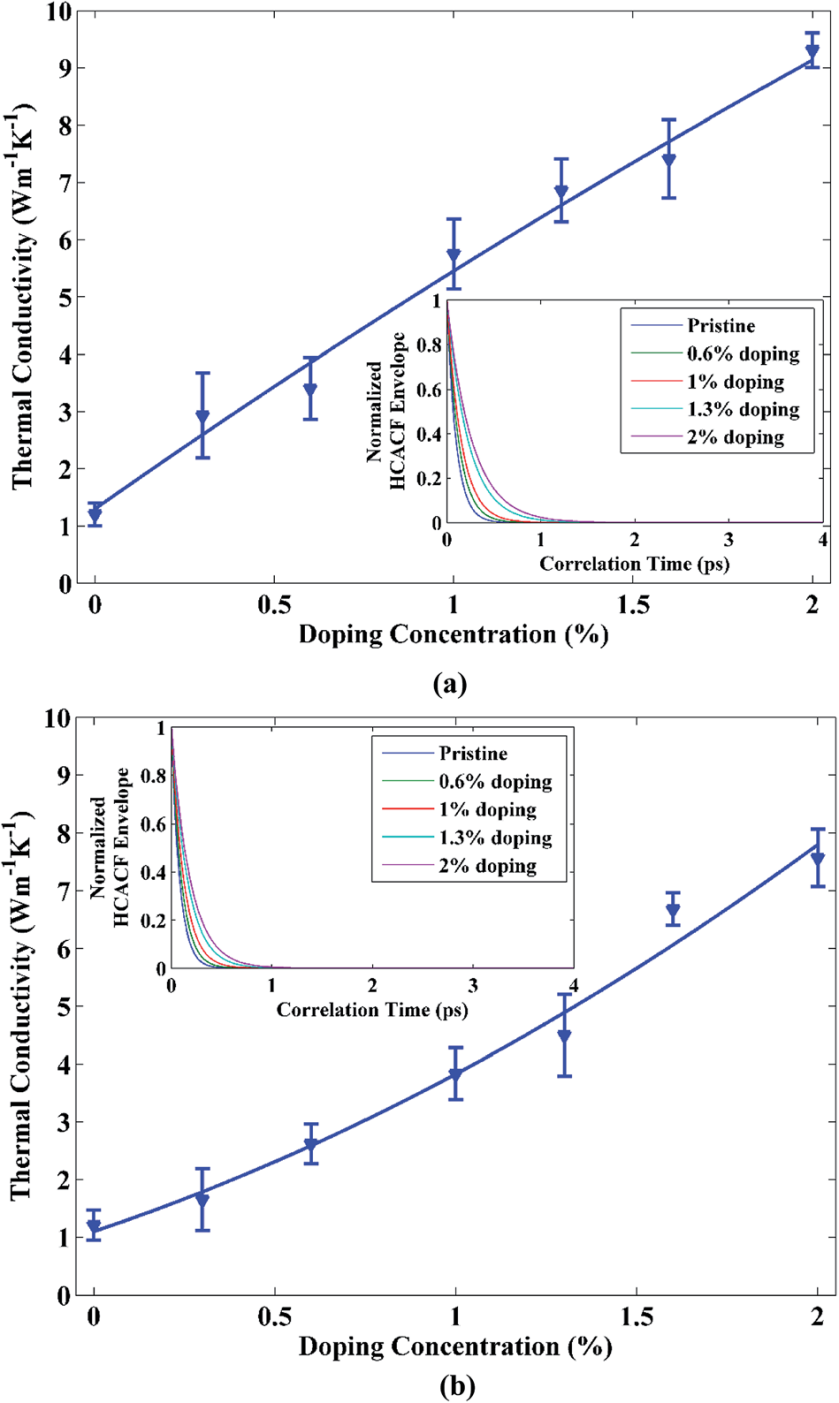
Thermal conductivity of 15 nm × 4 nm doped STNR (single doped) as a function of (a) carbon and (b) silicon doping concentration. The solid lines depict the numerically fitted curves through the data. The corresponding envelopes of normalized HCACF profiles for different doping concentrations are shown in the insets.

Our estimated thermal conductivity for 15 nm × 4 nm pristine STNR at room temperature is 1.204 ± 0.21 W m^−1^ K^−1^. Khan *et al.*^33^ reported the room temperature thermal conductivity for 10 nm × 3 nm sized zigzag STNR to be 0.95 ± 0.024 W m^−1^ K^−1^ by using EMD which is in good agreement with our result. Moreover, Cherukara *et al.*^19^ estimated the thermal conductivity value of 2.8 ± 0.2 W m^−1^ K^−1^ at 300 K for 80 nm × 80 nm zigzag stanene sheet and they predicted the lowering of this thermal conductivity value with nanostructuring. This also conforms well to our obtained result. On the other hand, using first principle calculations, Nissimagoudar *et al.*^47^ computed the room temperature thermal conductivity of zigzag stanene sheet to be 10.83 W m^−1^ K^−1^ and Peng *et al.*^17^ reported the room temperature stanene thermal conductivity to be 11.6 W m^−1^ K^−1^. The authors also expected further reduction in the thermal conductivity values with decreasing dimensionality and this is in accordance with our result as well. However, STNR doped with carbon and silicon exhibits thermal conductivity variation as shown in Fig. 2(a) and (b), respectively. For both the doping materials, the thermal conductivity of the STNR increases with the increase of doping concentration. The calculated room temperature thermal conductivities of doped STNR at 2% doping concentration of carbon and silicon are 9.31 ± 0.33 W m^−1^ K^−1^ and 7.57 ± 0.48 W m^−1^ K^−1^, respectively.

As STNR is doped with impurity, most of the high frequency phonons are localized due to the impurity centers.^48^ Therefore, the contribution of heat conduction from high frequency phonons is largely suppressed. As a result, the low frequency phonons with longer wavelengths play the dominant role in heat transport under these circumstances. Now, due to the low Debye temperature of pure stanene, there is an elevated scattering rate of high frequency phonons resulting in their low phonon group velocity and thus low thermal conductivity of pristine stanene.^17^ On the other hand, low frequency phonons in stanene have comparatively high group velocities and hence low scattering. Therefore, majority of thermal transport contribution in pristine stanene comes from these low frequency phonon modes.^17^ The impurity centers due to doping localize and suppress the high frequency phonons which have greater scattering rates. As a result, the weakly scattering low frequency phonon modes conducive to thermal conduction become more dominant. Hence, there is an overall improvement in the thermal conductivity of the carbon and silicon doped STNR. This fact is further illustrated by the HCACF profiles depicted in the insets of Fig. 2(a) and (b) for carbon and silicon doped structure, respectively. There is an enhanced localization hence suppression of high frequency phonons having greater scattering rates with increase in doping concentration. Consequently, the HCACF profiles decay at slower rates with the increasing doping concentration for both carbon and silicon doping. Slower decay rates of HCACF profile result in the calculation of higher thermal conductivity of doped STNR.

**Fig. 3:**
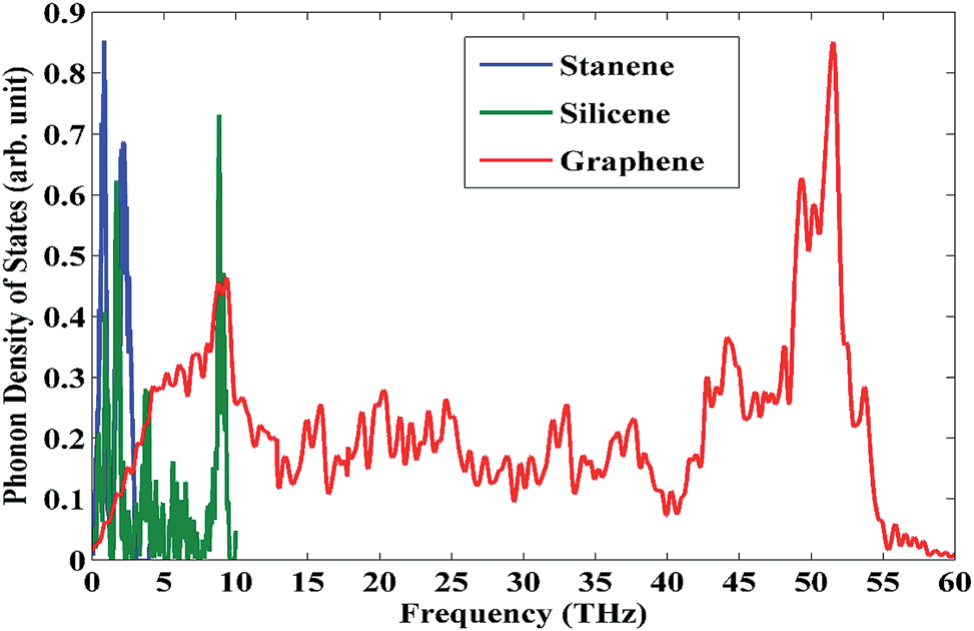
Phonon density of states for representative pristine stanene, silicene and graphene nanoribbons.

The thermal transport in the doped stanene can be further explained considering the phonon density of states (PDOS) for pristine stanene as well as its carbon and silicon counterparts graphene and silicene, respectively as shown in Fig. 3. The PDOS profiles of graphene and silicene both have large peaks at higher frequency regions (∼50 THz and ∼10 THz respectively) compared to that of stanene (∼2 THz). These peaks at high frequency regions for both graphene and silicene result in their much larger thermal conductivity values than that of stanene.^49,50^ Therefore, the incorporation of these comparatively high thermal conductivity materials into the low thermal conductivity nanostructure such as stanene would enhance the thermal transport property of the overall system.

**Fig. 4:**
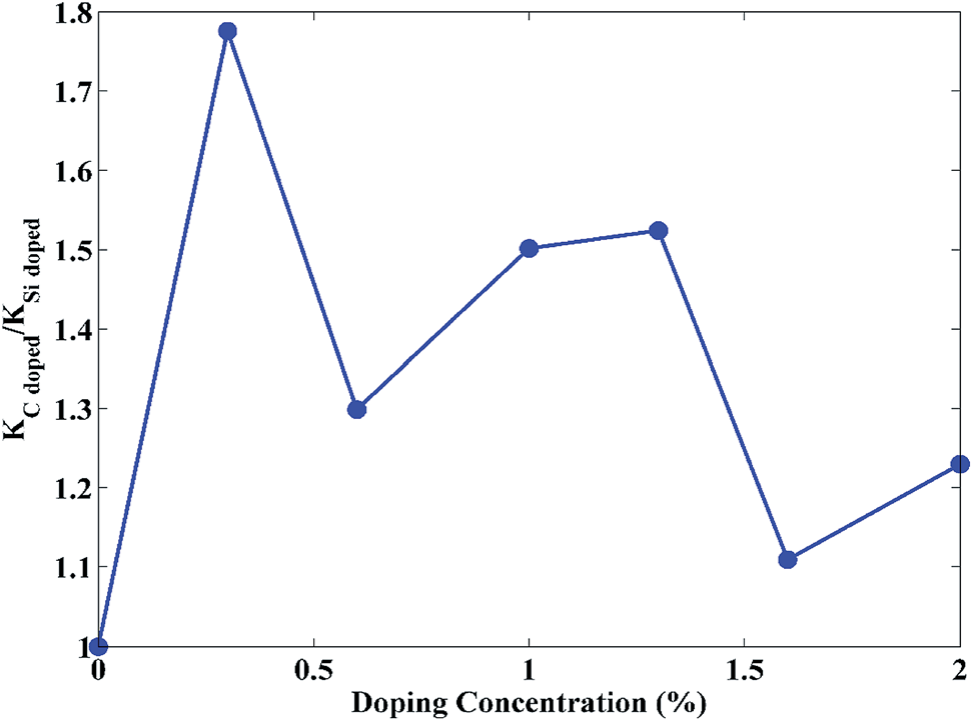
Ratio of thermal conductivity for carbon doped STNR (*K*_C doped_) and that of silicon doped STNR (*K*_Si doped_) as a function of doping concentration.

As can be seen from Fig. 4, the thermal conductivity of carbon doped STNR is higher than the silicon doped nanostructure since the ratio of thermal conductivity for carbon and silicon doped STNR is greater than one at all concentrations. This can be attributed to the mass effect of these elements. The carbon doped STNR has smaller average atomic mass than that of silicon doped STNR. Smaller average atomic mass results in higher Debye temperature which corresponds to higher value of thermal conductivity.^51^ This can be further explained from the fact that high atomic masses lower the sound velocity in materials thereby reducing the thermal conductivity.^52^ As a result, doping stanene with the heavier atom *i.e.* silicon has less increase in thermal conductivity compared to doping with the lighter atom *i.e.* carbon.

**Fig. 5:**
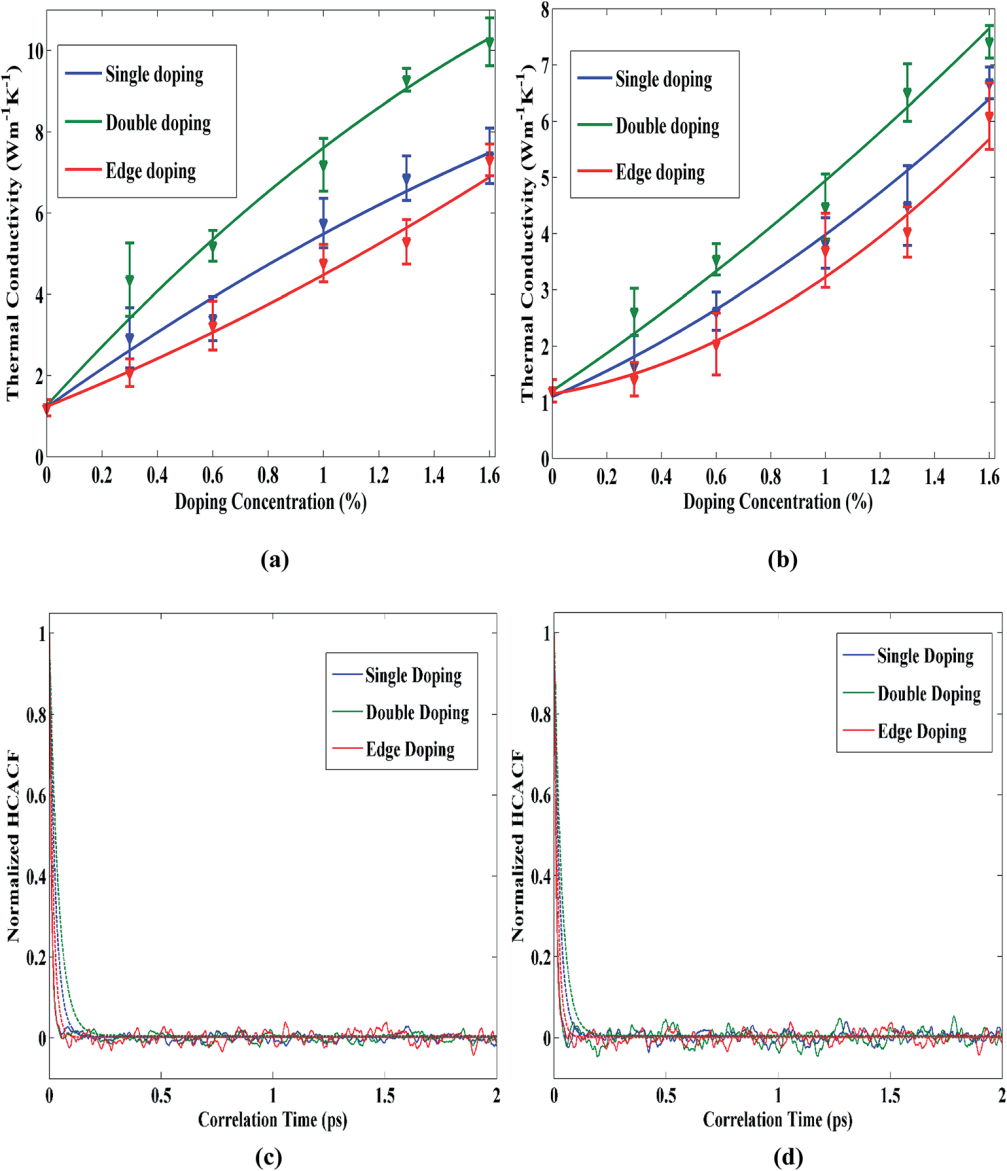
Thermal conductivity of 15 nm × 4 nm doped STNR as a function of (a) carbon and (b) silicon doping concentration for single, double and edge patterned doping. The solid lines represent the numerically fitted curves through the data. Normalized HCACF curves as well as their envelopes *versus* correlation time with 0.3% (c) carbon and (d) silicon doping for single, double and edge patterned doping at room temperature.

Next, we consider the impact of doping patterns on the thermal conductivity of STNR. Fig. 5(a) and (b) show the thermal conductivity variation of single, double and edge pattern doped STNR as a function of carbon and silicon doping concentration, respectively. The results suggest that the thermal conductivity of STNR increases with increased doping concentration for all three types of doping patterns. The thermal conductivity of double doped structure has higher value compared to other two patterns while of the remaining two patterns, single doping has greater thermal conductivity enhancement impact than edge doping.

In case of the double doping pattern, the doping centers act more like a molecule (*i.e.* C–C, Si–Si) doping center and the number of localized low frequency phonons is low.^53^ Hence, the delocalized low frequency phonons available for double doping pattern contribute to the large thermal conductivity enhancement.

For single doping pattern, the single doping centers cause degeneracy in the low frequency region around discrete single dope centers which results in localization of more low frequency phonon modes compared to double doping pattern.^53^ As a result, thermal conductivity enhancement due to low frequency modes in single doped structure is not so high as that of double doped STNR. Furthermore, since edge doping is a special case of single doping pattern, along with the enhanced localized low frequency phonon modes, edge dope centers additionally cause phonon edge scattering. This, in turn, limits its thermal conductivity enhancement impact in comparison with single and double doping patterns. For understanding this phenomenon further, Fig. 5(c) and (d) can be taken under consideration which depict the reasonable decay of HCACF profiles as well as their envelopes for single, double and edge doping patterns with carbon and silicon doping respectively. In both of these figures, it can be observed that the HCACF profile decays in the shortest time for edge doping pattern followed by single doping and double doping patterns respectively, thus substantiating the thermal conductivity variations found for these doping patterns.

**Fig. 6:**
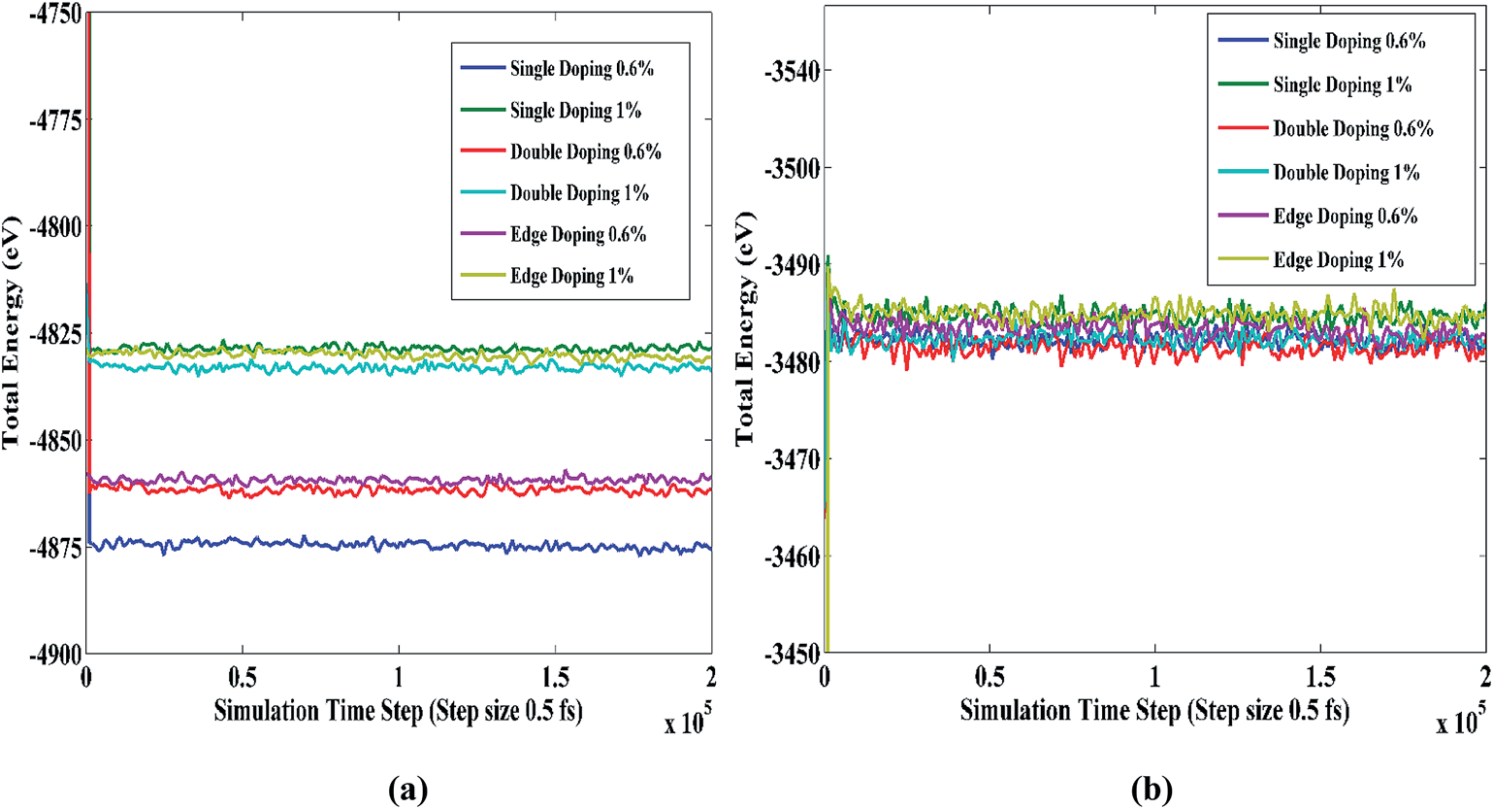
Total energy of different types of doped STNRs as a function of simulation time at room temperature for (a) carbon doping (b) silicon doping.

Fig. 6(a) and (b) depict the total energy during the simulation time for several STNR doping patterns at 0.6% and 1% carbon and silicon doping concentration, respectively. In both cases, it can be observed that the energy variations of the doped STNRs are negligible. This, in turn, implies that the STNR structures of various doping patterns with carbon and silicon dopants are energetically well stable.

**Fig. 7:**
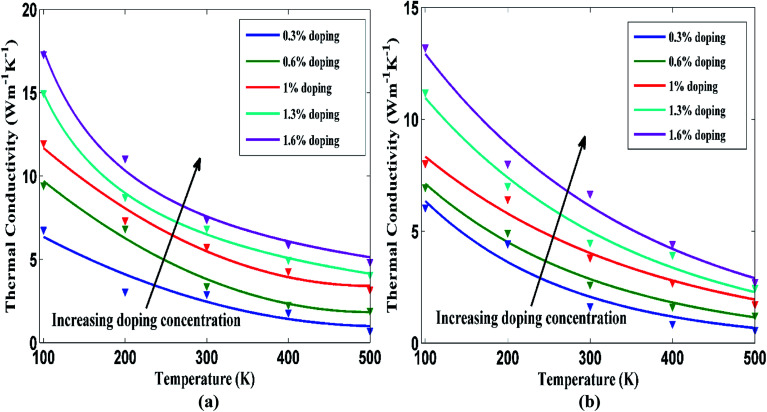
Thermal conductivity of 15 nm × 4 nm doped STNR as a function of temperature with various doping concentrations for (a) carbon and (b) silicon doping. The solid lines represent the numerically fitted curves through the data.

Next, the temperature dependence of thermal conductivity for doped STNR has also been investigated for different doping concentrations. Fig. 7(a) and (b) present the thermal conductivity of a 15 nm × 4 nm doped STNR with carbon and silicon atoms, respectively as a function of temperature for doping concentrations ranging from 0.3% to 1.6%. The thermal conductivity of STNR monotonically decays with increasing temperature for a specific doping concentration. This trend is in agreement with the studies of thermal conductivity for doped graphene by Goharshadi *et al.*^27^ This also conforms well to the results of Ye *et al.* where it is reported that the thermal conductivity of graphene nanoribbon (GNR) is reduced with increasing temperature due to significant decrease in relaxation time.^54^ Furthermore, Peng *et al.*,^17^ Cherukara *et al.*^19^ and Khan *et al.*^33^ also reported similar temperature dependence in thermal transport of pristine stanene nanostructures.

The thermal conductivity drooping characteristics with the increasing temperature at a particular doping concentration can be explained considering phonon–phonon anharmonic interaction or Umklapp scattering at an elevated temperature. At high temperature, Umklapp scattering becomes highly significant^55^ and the thermal conductivity is dominated by the highly energized thermally excited phonons. As a result, thermal conductivity decreases with the increase of temperature. It is observed that thermal conductivity maintains an inverse relation *T*^−1^ with temperature at the beginning but at much higher temperature values, this functional relation is no longer applicable. At sufficiently high temperatures, enhanced anharmonic interactions between the two acoustic phonon modes are accompanied by higher order scattering process^56–58^ which results in non-linear thermal resistivity. Similar decaying characteristics of thermal conductivity with the increased temperature are observed for other doping concentrations while the curves shift upward with the increasing doping concentration. This is in agreement with the earlier observation that for a specific temperature, the thermal conductivity of doped stanene increases with the increasing doping concentration.

**Fig. 8:**
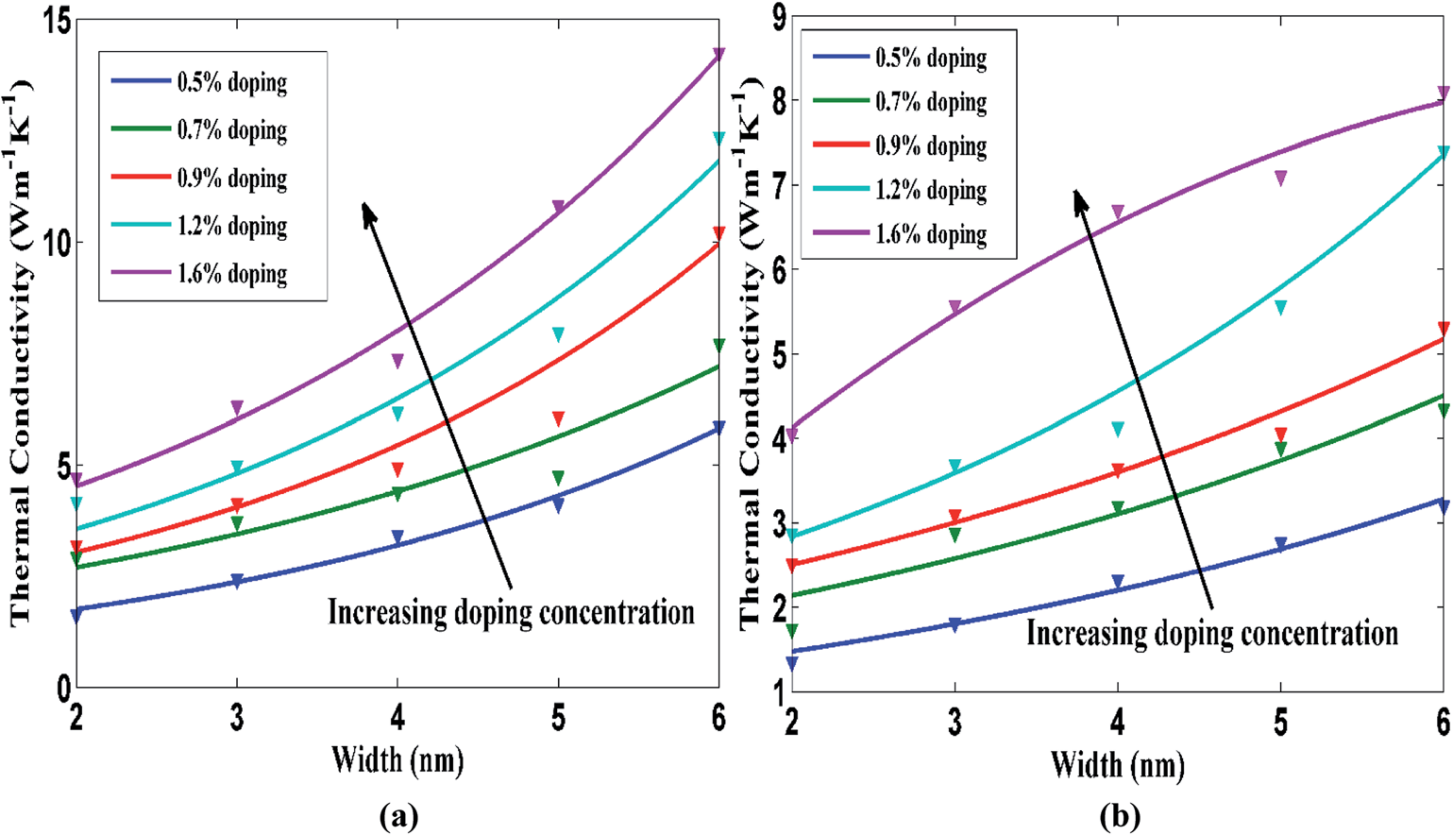
Room temperature thermal conductivity of doped STNR as a function of the nanoribbon width with varying doping concentrations for (a) carbon and (b) silicon doping. The nanoribbon length is fixed at 15 nm. The solid lines represent the numerically fitted curves through the data.

The width dependence of STNR for different carbon and silicon doping concentration has been studied as depicted in Fig. 8(a) and (b), respectively. The figures display the thermal conductivity change of STNR with respect to the nanoribbon width ranging from 2 nm to 6 nm for carbon and silicon doping concentrations of 0.5%, 0.7%, 0.9%, 1.2% and 1.6% while length of the ribbon is kept constant at 15 nm. The thermal conductivity increases with the increasing width for a specific doping concentration. This result is in line with the investigation on the width dependence of thermal conductivity by Khan *et al.*^33^ for pristine stanene, by Sevik *et al.*^59^ for pristine hexagonal boron nitride nanoribbon as well as by Cao^60^ and Yang *et al.*^61^ for graphene nanoribbon. Ye *et al.* also found a decreasing trend of GNR thermal conductivity with the reduction of width and attributed it to more intensified boundary scattering with smaller nanoribbon width.^54^ The set of curves in Fig. 8(a) and (b) drift upwards for increasing doping concentrations.

Two factors namely, edge localized phonon effect or boundary scattering effect and anharmonic phonon–phonon scattering effect need to be considered to provide the better insight of the width effect on STNR thermal conductivity since both the factors adversely affect the thermal conductivity. As the doped STNR width increases, the impact of boundary scattering is reduced resulting in the rise of thermal conductivity. Moreover, with the increase of the ribbon width, the probability of Umklapp scattering is heightened as the number of available phonons increases.

As these two processes contend with each other, the thermal transport characteristics are regulated by the more dominant one. For comparatively narrow STNRs which is the case of our study, the lowering of boundary scattering effect in wider ribbon is more dominant than the intensified Umklapp scattering effect and therefore thermal conductivity rises with the increase in ribbon width.^62–65^

## 4. Conclusions

We investigated the impact of carbon and silicon doping concentration as well as doping patterns namely single doping, double doping and edge doping on the thermal transport characteristics of STNR employing equilibrium molecular dynamics simulation in this study. Thermal conductivity of STNR follows an increasing trend with the increasing doping concentration, for both carbon and silicon dopants. This can be attributed to the localization of the high frequency phonon modes having greater scattering rates allowing the weakly scattering low frequency phonon modes to contribute to the thermal conductivity enhancement. Doped STNR with carbon atoms shows higher thermal conductivity than silicon doping owing to the mass difference of carbon and silicon *i.e.* carbon being lighter than silicon. Double doping pattern, among the considered three patterns, is found to be the most influential in the thermal transport improvement of STNR as this pattern causes the least amount of low frequency phonon modes localization. On the other hand, edge doping pattern yields the least amount of thermal conductivity variation. We also investigate the thermal conductivity as a function of temperature and width of the ribbon. Both carbon and silicon doped STNR shows a decaying thermal conductivity with the increasing system temperature at a particular doping concentration due to high frequency phonon–phonon scattering. Moreover, the doped nanoribbon thermal conductivity continues to increase with the increasing nanoribbon width since the boundary scattering in doped STNR decreases as the width increases and the Umklapp scattering process is least dominant for the range of nanoribbon width considered in this study. Our results would provide valuable insight in realizing the possible application of doped stanene nanostructures in thermoelectric and nanoelectronic devices.

## Conflicts of interest

There are no conflicts to declare.

## Supplementary Material
